# Impaired B Cell Recall Memory and Reduced Antibody Avidity but Robust T Cell Response in CVID Patients After COVID-19 Vaccination

**DOI:** 10.1007/s10875-023-01468-w

**Published:** 2023-03-17

**Authors:** Sophie Steiner, Tatjana Schwarz, Victor M. Corman, Lara M. Jeworowski, Sandra Bauer, Christian Drosten, Carmen Scheibenbogen, Leif G. Hanitsch

**Affiliations:** 1 Institute of Medical Immunology, Charité - Universitätsmedizin Berlin, Corporate Member of Freie Universität Berlin and Humboldt Universität Zu Berlin, Campus Virchow, Augustenburger Platz 1/Südstraße 2, 13353 Berlin, Germany; 2 Berlin Institute of Health, Berlin, Germany; 3 Institute of Virology, Charité - Universitätsmedizin Berlin, Corporate Member of Freie Universität Berlin and Humboldt-Universität Zu Berlin, German Centre for Infection Research (DZIF), Associated Partner, Charitéplatz 1, 13353 Berlin, Germany; 4 Labor Berlin-Charité Vivantes GmbH, Berlin, Germany; 5 Berlin Institute of Health at Charité - Universitätsmedizin Berlin, BIH Center for Regenerative Therapies (BCRT), Charitéplatz 1, Berlin, Germany

**Keywords:** CVID, COVID-19, vaccination, memory, T cell response, antibody response

## Abstract

**Purpose:**

Humoral and cellular immune responses were described after COVID-19 vaccination in patients with common variable immunodeficiency disorder (CVID). This study aimed to investigate SARS-CoV-2-specific antibody quality and memory function of B cell immunity as well as T cell responses after COVID-19 vaccination in seroresponding and non-responding CVID patients.

**Methods:**

We evaluated antibody avidity and applied a memory B cell ELSPOT assay for functional B cell recall memory response to SARS-CoV-2 after COVID-19 vaccination in CVID seroresponders. We comparatively analyzed SARS-CoV-2 spike reactive polyfunctional T cell response and reactive peripheral follicular T helper cells (pT_FH_) by flow cytometry in seroresponding and non-seroresponding CVID patients. All CVID patients had previously failed to mount a humoral response to pneumococcal conjugate vaccine.

**Results:**

SARS-CoV-2 spike antibody avidity of seroresponding CVID patients was significantly lower than in healthy controls. Only 30% of seroresponding CVID patients showed a minimal memory B cell recall response in ELISPOT assay. One hundred percent of CVID seroresponders and 83% of non-seroresponders had a detectable polyfunctional T cell response. Induction of antigen-specific CD4^+^CD154^+^CD137^+^CXCR5^+^ pT_FH_ cells by the COVID-19 vaccine was higher in CVID seroresponder than in non-seroresponder. Levels of pT_FH_ did not correlate with antibody response or avidity.

**Conclusion:**

Reduced avidity and significantly impaired recall memory formation after COVID-19 vaccination in seroresponding CVID patients stress the importance of a more differentiated analysis of humoral immune response in CVID patients. Our observations challenge the clinical implications that follow the binary categorization into seroresponder and non-seroresponder.

**Supplementary Information:**

The online version contains supplementary material available at 10.1007/s10875-023-01468-w.

## Introduction

Coronavirus disease (COVID-19) is caused by severe respiratory syndrome coronavirus-2 (SARS-CoV-2), and has caused more than 500 million infections and over 6 million deaths worldwide since its emerging in late 2019 [1].

Patients with common variable immunodeficiency disorder (CVID), the most frequent clinically relevant primary immunodeficiency, are at higher risk for COVID-19-associated hospitalization and mortality [2] as well as for increased risk of prolonged or recurrent (breakthrough) SARS-CoV-2 infections [3]. Vaccination is considered to be the most effective and safest prophylactic measure in patients with primary immunodeficiency including genetically defined inborn errors of immunity (IEI) [4]. Cumulative data on COVID-19 vaccine response from more than 1500 patients with IEI have been reported with CVID being the most frequent underlying immunodeficiency [5]. Findings on humoral immune response after COVID-19 vaccination are variable with frequencies of seroresponding CVID patients ranging from 20 to 95% [6–12]. A positive T cellular immune response was reported in 46–83% of CVID patients [8, 10–15]. Reasons for the observed variability might include differences in methodology and different vaccination regimens as well as clinical, immunological, and genetic heterogeneity of CVID patients.

More importantly, the detection of specific antibodies after COVID-19 vaccination raises important questions on the quality and longevity of the humoral immune response, since the seroconversion state has potential clinical implications for treatment in SARS-CoV-2-infected CVID patients. Functional assessments of humoral immunity already revealed that CVID patients express lower neutralizing antibody levels than healthy individuals [8–11, 15]. Avidity of generated antibodies was analyzed in two recent studies, reporting similar levels of antibody avidity in CVID patients and healthy individuals 4 weeks after second COVID-19 vaccination but without significant increase after more than two vaccinations [9, 16]. Detailed characterizations regarding B cell memory formation are limited and suggest an atypical memory formation [17]. Without affecting seroconversion rates, booster vaccination in CVID patients was shown to further increase antibody levels in some seroresponder [9, 18], while effects of boosting on specific T cell immunity is variable [9, 10].

In the present study, we evaluated antibody avidity and functional B cell recall memory responses to SARS-CoV-2 vaccination in CVID seroresponders. In addition, specific polyfunctional T cell response and the generation of SARS-CoV-2 specific follicular T helper cells (T_FH_) were assessed by flow cytometry in CVID seroresponder and non-seroresponder. All included CVID patients had a previously documented impaired specific antibody response to conjugated pneumococcal vaccination.

## Methods

### Study Subjects

Samples of 16 CVID patients before first and after the second SARS-CoV-2 vaccination were collected from the outpatient clinic for immunodeficiencies at the Institute for Medical Immunology, Charité Universitätsmedizin Berlin. All CVID patients were adults and diagnosed according to the criteria defined by the European Society for Immune Deficiency (ESID) [19]. CVID patients had an impaired vaccine response to pneumococcal conjugate vaccine (specific IgG antibodies below protective levels or low specific antibodies without increase after vaccination). Samples of 8 healthy controls (HC) before and after the second dose of SARS-CoV-2 vaccination were collected from laboratory employees at Charité Universitätsmedizin Berlin. All samples were collected between June and October 2021. During this period, SARS-CoV-2 B.1.617.2 (Delta) was the most predominant strain in Germany. All CVID patients and HC were infection naïve with no clinical history of SARS-CoV-2 infection, expressing negative spike antibodies before first vaccination (Table 2) and remaining seronegative for nucleocapsid (NP) after vaccination, to address possible infections between sampling time points (Supplementary Table S2).

### Sample Preparation

Serum and heparinized whole blood was collected at a median of 133 days for CVID seroresponder (IQR: 24) and 128 days for CVID non-seroresponder (IQR: 34) after second COVID-19 vaccination. Samples from healthy individuals were collected at 32 days (IQR: 5) after second COVID-19 vaccination. Peripheral blood mononuclear cells (PBMCs) were isolated by density gradient centrifugation over Pancoll (PAN-Biotech, Germany) using Leucosep tubes (Greiner Bio-One). PBMCs were cryopreserved and stored in liquid nitrogen.

### SARS-CoV-2 Antibody Serology

SARS-CoV-2 spike serum IgG against the S1 domain was assessed by ELISA according to the manufacturer’s instructions (Euroimmun Medizinische Labordiagnostika AG, Lübeck, Germany) using fully automated Euroimmun Analyzer I (Euroimmun Medizinische Labordiagnostika AG, Lübeck, Germany). To confirm results obtained by ELISA, a microarray-based multiparametric immunoassay for detection of IgG antibodies against SARS-CoV-2 spike and NP (SeraSpot® Anti-SARS-CoV-2 IgG, Seramun Diagnostica GmbH, Heidesee, Germany) was applied.

### IgG Avidity Assay

To measure avidity of SARS-CoV-2 spike IgG antibodies, serum samples were analyzed by a modified SARS-CoV-2-S1 ELISA (Euroimmun) [20]. Serum samples were diluted 1:101 with sample buffer and incubated on plates pre-coated with recombinant SARS-CoV-2 spike (S1) proteins. After incubation for 1 h at 37 °C, wells were washed and 200 µL urea (5.5 M.) or 200 µL phosphate-buffered saline (PBS) was added to the plates and incubated for 10 min at 37 °C. After a washing step, conjugate and substrate were added according to the manufacturer’s instructions. OD was detected at 450 nm, and the relative avidity index was calculated by dividing the observed OD of the urea-treated sample by that of the PBS-treated sample, multiplied by 100 [20].

### SARS-CoV-2 Interferon-Gamma Release Assay (IGRA)

IGRA (Euroimmun) for quantitative IFNγ release by SARS-CoV-2-specific T cells following second dose SARS-CoV-2 vaccine was performed according to the manufacturer’s instructions. In summary, 500 µl heparinized whole blood was added to three stimulation tubes coated with specific SARS-CoV-2 S1 peptide pool, mitogen control, and uncoated blank, respectively. Blood was incubated for 24 h at 37 °C, 5% CO_2_. Collected plasma was stored at − 20 °C until analysis by Quan-T-Cell ELISA (Euroimmun). According to manufacturer values ≥ 200 IU/ml are positive, values between 100 and 200 IU/ml are considered borderline.

### T Cell Phenotyping for SARS-CoV-2 Spike Reactive T Cells by Flow Cytometry

For each experimental approach, patient and control samples before and after vaccination were simultaneously assessed. Cryopreserved PBMCs were thawed and rested for 24 h in IMDM/10% FCS/1% P/S at 37 °C, 5% CO_2_. Stimulation was performed with 1 µg/ml of SARS-CoV-2 S peptide pools for N- and C-terminal domains (PM-WCPV-S-1, JPT Peptide Technologies GmbH, Berlin). Superantigen staphylococcal enterotoxin B (SEB) was used (3 µg/ml) as positive and DMSO as background control. Secretion inhibitor brefeldin A (BFA) (15 µg/ml) was added to each condition after 2 h. Stimulation continued for 16 h. Cells were washed and extracellular markers for anti-human CCR7 AF488, CD45RA PE-Cy7, and Live/Dead Fixable Blue stained for 30 min at 37 °C, 5% CO_2_. After repeated washing, fixation/permeabilization buffer was applied (FoxP3 transcription factor staining buffer set, eBioscience) and incubated for 30 min at 4 °C. Intracellular staining was performed for anti-human CD3 BV650, CD4 PerCp-Cy5.5, CD8 BV510, CD137 PE, CD154 BV421, IL-2 APC, IFNγ BV605, TNFα AF700, and CXCR5 PE-Dazzle (Supplementary Table S3) for 30 min at 4 °C. CytoflexLX flow cytometer and FlowJo software version 10.6.2 were used for analysis. Unspecific activation was excluded by subtracting the background signal (DMSO only) from the peptide and SEB activated samples. A positive T cell response was defined as CD154^+^CD137^+^CD4^+^ T cells > 0.005% within total CD4^+^ T cell population and 20% above the background. Boolean combination gating was used for analysis of single and polyfunctional cytokine producing T cell subsets.

### Memory B Cell ELISPOT Assay

Cryopreserved PBMCs were thawed and seeded in a 6-well plate at a concentration of 4 × 10^6^ cells in 3 ml RPMI/10% FCS/1% P/S (culture medium) per well in the presence of 5% CO_2_ at 37 °C. B cell proliferation was induced by the protocol from Crotty et al. [21] with 6 µg/ml CpG, 100 ng/ml of Pokeweed mitogen (PWM), staphylococcus aureus Cowan (SAC) (1:10 000), and 50 μM β-Mercaptoethanol for 7 days (called SAC protocol hereafter).

For the detection of antibody secreting cells (ASC) in HC and seroresponsive CVID patients after the second dose of SARS-CoV-2 vaccine, ELISPOT assay (enzyme-linked immuno spot assay) was performed with expanded cells after the 7-day cell culture. 96-well MultiScreen Filter Plates (Merck Millipore) were coated overnight (ON) at 4 °C with 1 µg/ml stabilized trimeric spike protein SARS-CoV-2 (wild type Excell Gene), as well as 1.2 μg/ml goat anti-human IgG (Jackson ImmunoResearch), serving as positive control. PBS was applied as negative control to exclude unspecific antibody binding.

Expanded cells were plated in the 96-well MultiScreen Filter Plates in duplicate at dilutions of 2.5 × 10^5^/100 µl (IgG) and 1 × 10^6^/100 µl for SARS-CoV-2 S protein, 6.25 × 10^3^/100 µl for IgG positive controls and incubated for 6 h. Wells were then thoroughly washed six times with PBS supplemented with 1% bovine serum albumin (BSA) and 0.05% Tween. 100 μl/well, goat anti-human IgG-HRP (1:500) (Invitrogen) secondary antibody was applied and incubated ON at 4 °C. Afterwards, wells were washed three times with PBS. Substrate buffer (0.3 M sodium acetat solution, 0.2 M acetic acid solution, Auqa dest., pH = 5.0), 3-amino-9-ethyl-carbazole (AEC)-dimethylformamide (DMF) solution (1:30) and 3% H_2_O_2_ (1:100)) was applied to reach spot development. AID ELISPOT reader and AID ELISPOT 7.0 iSpot software were used for analysis. Results were manually verified to exclude artificial spots and multiple counting.

### B Cell Phenotyping by Flow Cytometry

FACS analysis of B cell subsets was performed on PBMCs of day 0 (ex vivo) and on day 7 after cell culture (in vitro). Cells were incubated with a LIVE/DEAD fixable Aqua (Thermo Fisher) for 30 min at RT. Extracellular staining with fluorescently conjugated antibodies CD3 PB, CD19 PE-Cy7, CD21 PE, CD24 PerCp-Cy5.5, CD27 FITC, CD38 AF700, IgM APC, and IgD APC-Cy7 (Supplementary Table S4) for 30 min at 4 °C was performed. CytoflexLX flow cytometer and FlowJo software version 10.6.2 were used for analysis. Gating for B cell phenotyping was performed according to EUROClass classification (Supplementary Table S5, Supplementary Fig. S1) [22].

### Statistical Analyses

Unpaired comparisons across multiple groups were performed using the Kruskal–Wallis test with Dunn’s post-test for multiple comparisons to find significant differences among multiple investigated groups. If a significance was detected, two-tailed Mann–Whitney *U* test was performed for unpaired comparisons across two groups. In order to analyze responses before and after vaccination, the Wilcoxon matched pairs signed-rank test was applied for paired comparisons within a group. Correlation analyses were performed using Spearman’s rank correlation coefficient. Continuous variables are shown as median and interquartile range (IQR). A *p*-value of < 0.05 was considered statistically significant. GraphPad Prism version 9.3.1 was used for statistical analyses.

## Results

### Study Cohort Characteristics

Ten seroresponding (CVID R), 6 non-responding (CVID NR) CVID patients, and 8 healthy controls (HC) were analyzed unvaccinated and after second COVID-19 vaccination. Seroresponse was defined according to manufactures instruction by a ratio of  > 1,1 for spike-specific SARS-CoV-2 IgG antibody response in Euroimmun ELISA (Table 1). Data from SeraSpot analysis confirmed humoral immune response to spike (S1, S full, and RBD) in CVID R (Supplementary Table S2). Basic immunological parameters included IgG, IgA, IgM, CD3^+^, CD4^+^, CD8^+^, and CD19^+^ cells as well as NK cells and T and B cell subsets (see Table 2) and showed no significant differences between the CVID groups. In addition, clinical characterization included non-infectious (immune cytopenia, autoimmunity, lympho-proliferation, granulomatous lymphocytic interstitial lung disease) and infectious manifestations (recurrent pneumonia and bronchiectasis) which showed no significant differences (see Table 3). Age and gender were similar in CVID R and CVID NR (median age 57 years).

**Table 1:** Serological data of anti-SARS-CoV-2 spike IgG in CVID patients and healthy controls (EUROIMMUN ELISA©)

	Before COVID-19 vaccination		After COVID-19 vaccination	
	IgG OD ratio	Result	IgG OD ratio	Result
CVID R 1	0.13	Negative	6.48	Reactive
CVID R 2	0.16	Negative	3.86	Reactive
CVID R 3	0.13	Negative	2.88	Reactive
CVID R 4	0.1	Negative	1.94	Reactive
CVID R 5	0.14	Negative	2.82	Reactive
CVID R 6	0.12	Negative	3.21	Reactive
CVID R 7	0.14	Negative	7.75	Reactive
CVID R 8	0.14	Negative	3.56	Reactive
CVID R 9	0.9	Negative	3.04	Reactive
CVID R 10	0.28	Negative	2.24	Reactive
CVID NR 1	0.14	Negative	0.14	Negative
CVID NR 2	0.15	Negative	0.66	Negative
CVID NR 3	0.14	Negative	0.28	Negative
CVID NR 4	0.25	Negative	0.23	Negative
CVID NR 5	0.14	Negative	0.48	Negative
CVID NR 6	0.23	Negative	0.15	Negative
HC-1	0.16	Negative	7.67	Reactive
HC-2	0.12	Negative	7.4	Reactive
HC-3	0.12	Negative	6.42	Reactive
HC-4	0.19	Negative	6.85	Reactive
HC-5	0.08	Negative	7.36	Reactive
HC-6	0.09	Negative	7.22	Reactive
HC-7	0.09	Negative	8.12	Reactive
HC-8	0.08	Negative	8.3	Reactive

*COVID-19*, coronavirus disease 2019; *CVID*, common variable immunodeficiency disorder; *HC*, healthy control; *NR*, non-seroresponder; *OD*, optical density; *R*, seroresponder

**Table 2:** Description of immunological parameter before COVID-19 vaccination comparing CVID seroresponder with CVID patients that failed to mount specific antibodies after completed COVID-19 vaccination

	IgG in g/L prior to IgRT	IgA in g/L	IgM in g/L	CD4^+^ (cell count per nl)	CD8^+^ (cell count per nl)	CD19^+^ (cell count per nl)	NK (cell count per nl)	Naïve CD4^+^ CD45RA^+^ in % of CD4^+^	Naïve Bc in % of CD19^+^	MZ-like Bc in % of CD19^+^	IgM + MBC in % of CD19^+^	CS MBC in % of CD19^+^	Transitional Bc in % of CD19^+^	Activated Bc in % of CD19^+^	CS PB in % of CD19^+^
Normal range	7–16	0.7–4.0	0.4–2.3	0.5–1.2	0.3–0.8	0.1–0.4	0.1–0.4	> 15	42.6–82.3	7.4–32.5	–	6.5–29.1	0.6–3.4	0.9–7.6	0.4–3.6
CVID R 1	1.30	0.06	0.11	0.59	0.72	0.96	0.39	10	71.9	4.3	0.3	0.5	8.7	12.6	0
CVID R 2	2.66	< 0.1	0.06	0.41	0.14	0.14	0.09	34	67.3	14.5	1.4	0.5	2.4	13.2	0.1
CVID R 3	< 0.3	0.06	0.05	0.57	0.61	0.28	0.09	21	86.2	5.7	0.3	1	4	1.6	0
CVID R 4	< 0.3	< 0.1	0.05	0.41	0.36	0.22	0.28	10	81.3	12.2	0.3	0.6	3.5	1.5	0
CVID R 5	2.00	0.06	0.1	0.55	0.23	0.07	0.06	6	71.4	3.5	0.3	1.3	11.4	12.3	0.1
CVID R 6	1.86	0.14	0.15	0.37	1.23	0.22	0.13	24	76.9	8.4	0.6	2.7	1.6	7.7	0.3
CVID R 7	3.70	0.59	0.4	0.69	0.34	0.24	0.07	62	79.8	7.6	0.4	0.8	1.7	9.1	0.1
CVID R 8	3.00	0.04	0.16	0.55	0.33	0.37	0.13	27	56.51	31.73	2.36	1.78	2.47	6.06	0.89
CVID R 9	0.77	< 0.1	0.05	0.26	0.76	0.14	0.41	6	53.7	9	3.8	0.7	9.2	21.6	0.8
CVID R 10	1.69	0.06	0.28	0.33	0.5	0.12	0.07	15	64.4	12.9	1.9	1.6	0.5	18.7	0
Median values CVID R 1–10	**1.78**	**0.06**	**0.13**	**0.48**	**0.43**	**0.22**	**0.11**	**18**	**71.65**	**8.7**	**0.5**	**0.9**	**2.985**	**10.7**	**0.1**
CVID NR 1	1.66	0.06	0.15	0.32	0.33	0.19	0.1	21	76	3.5	2.5	0.2	6.6	3.3	0
CVID NR 2	3.60	0.25	0.18	0.43	0.23	0.06	0.02	5	70.9	4.7	0	1.3	8	14.7	0.5
CVID NR 3	0.33	0.06	0.05	0.5	0.28	0.16	0.26	21	79.3	11.2	0.6	0.8	1.6	4.8	0.4
CVID NR 4	< 0.30	0.06	0.05	0.64	0.62	0.04	0.05	47	81.4	7.3	1.1	0.3	4.2	4.1	0
CVID NR 5	< 0.3	< 0.1	2.2	0.84	0.44	0.38	0.11	60	59.3	33.8	0	0.6	0.7	5.3	0.1
CVID NR 6	< 0.3	< 0.1	0.05	0.93	0.55	0.08	0.05	2	67.5	8.8	2.6	1.1	1.8	15.8	0
Median values CVID NR 1–6	**0.32**	**0.08**	**0.1**	**0.57**	**0.385**	**0.12**	**0.08**	**21**	**73.45**	**8.05**	**0.85**	**0.7**	**3**	**5.05**	**0.05**
*p*-value* CVID R vs. CVID NR	0.21	0.52	0.94	0.30	0.54	0.21	0.17	0.94	0.87	0.74	0.99	0.28	0.73	0.56	0.80

Values are in bold to highlight that this line is a summary of the group (CVID R and CVID NR respectively)

*Bc*, B cell; *CS*, class-switched; *CVID*, common variable immunodeficiency disorder; *g/L,* grams per liter; *HC,* healthy control; *IgRT*, immunoglobulin replacement therapy; *MBC*, memory B cells; *MZ*, marginal zone; *nl*, nanoliters; *NK*, natural killer cells; *NR*, non-seroresponder; *OD*, optical density; *PB*, plasmablasts; *R*, seroresponder; bolded values = median; *p*-value calculated using Mann–Whitney *U* test

**Table 3:** Clinical characterization of CVID seroresponder (CVID R) and CVID non-seroresponder (CVID NR) before COVID-19 vaccination in % of affected patients

	Immune cytopenia	Autoimmunity	Splenomegaly/lympho-proliferation	GLILD	Bronchiectasis	Rec. pneumonias	Immuno-suppression	Genetic diagnostics
CVID R	10%	20%	60%	10%	0%	30%	0	Negative in all patients
CVID NR	33%	0%	100%	0%	33%	50%	0	Negative in all patients
*p*-value	0.52	0.5	0.23	1	0.125	0.61	1	

*CVID*, common variable immunodeficiency disorder; *GLILD*, granulomatous lymphocytic interstitial lung disease; *NR*, non-seroresponder; *R*, seroresponder; *rec*., recurrent

### Impaired SARS-CoV-2 Spike Antibody Response in CVID Patients

**Fig. 1:**
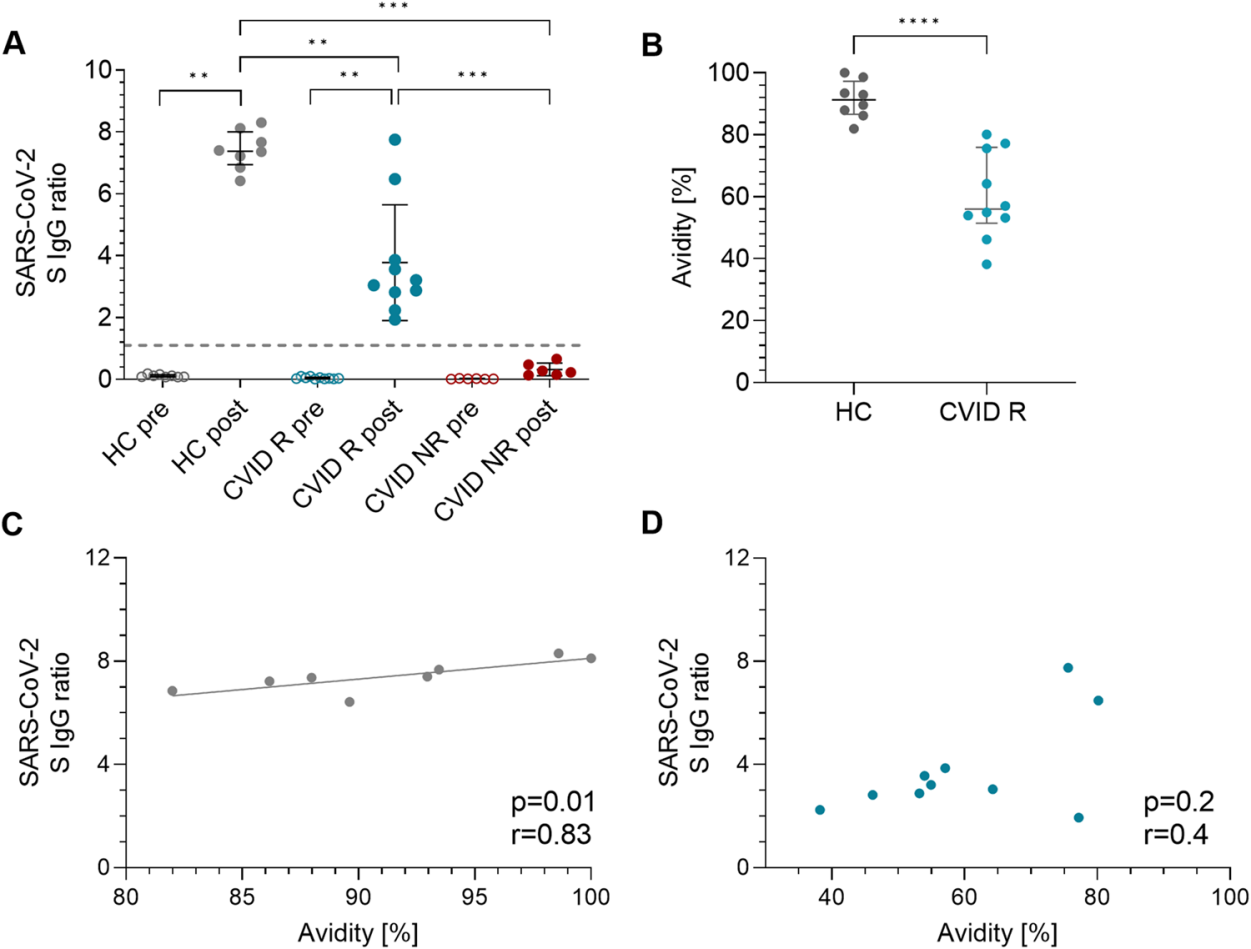
SARS-CoV-2 IgG antibody serology and avidity in COVID-19 vaccinated CVID patients and HC. **A** Serum IgG against the N-terminal domain of SARS-CoV-2 spike protein (EUROIMMUN Anti-SARS-CoV-2 ELISAs). Results displayed as OD ratio of the control or patient sample over the OD of a calibrator. Ratio < 0.8 = negative. Ratio ≥ 0.8 to < 1.1 = borderline. Ratio ≥ 1.1 = positive (dashed line). **B** SARS-CoV-2 IgG antibody avidity in HC and CVID R patients assessed by SARS-CoV-2 RBD avidity ELISA. Median and interquartile range (IQR) are indicated. Unpaired comparisons across two groups were done by two-tailed non-parametric Mann–Whitney *U* test; paired comparisons within a group were done using the Wilcoxon matched pairs signed-rank test; *p* ≤ 0.05; **: *p* ≤ 0.01; ***: *p* ≤ 0.001; ****: *p* ≤ 0.0001. **C**–**D** Correlation of SARS-CoV-2 S IgG with avidity in HC (**C**) and CVID R patients (**D**). Correlation analysis was performed using Spearman’s rank correlation coefficient

Spike-specific SARS-CoV-2 IgG antibody response was analyzed in two different systems (ELISA and SeraSpot). ELISA indicated a significant increase of spike-specific SARS-CoV-2 IgG antibodies after COVID-19 vaccination in HC (IgG: *p* = 0.008) and CVID R patients (IgG: *p* = 0.002), but antibody levels were significantly lower in CVID R patients compared to HC (IgG: *p* = 0.002) (Fig. 1A). Data from SeraSpot analysis indicated negativity for NP, which is not induced by the spike-based COVID-19 vaccination but by a previous SARS-CoV-2 infection (Supplementary Table S2).

### SARS-CoV-2 Spike Antibody Avidity Is Significantly Diminished in Seroresponding CVID Patients

SARS-CoV-2 spike antibody avidity of CVID R patients was significantly lower than in HC (*p* < 0,001; Fig. 1B). Avidity correlated with levels of SARS-CoV-2 spike IgG in HC (*p* = 0.01; *r* = 0.833, Fig. 1C) but not in CVID R patients (*p* = 0.2; *r* = 0.45 Fig. 1D).

### Formation of B Cell Memory Is Impaired in CVID Patients Despite the Presence of Circulating Antibodies

It is unknown whether seroconversion in CVID patients could also result in the development of a functional B cell memory. Here, we aimed to study the functional memory B cell (MBC) response after COVID-19 vaccination in CVID R patients and HC. ELISPOT results were analyzed in combination with B cell subsets (for gating see Supplementary Fig. S1). For comparability, data was calculated per B cell and PB proportion within CVID patients and HC. IgG secreting cells, detected in the ELISPOT assay, were calculated per 10,000 PBs and 10,0000 MBC which were used on day 0 for in vitro stimulation.

#### Memory B Cell and Plasmablast Phenotype in Seropositive CVID Patients and HC

**Fig. 2:**
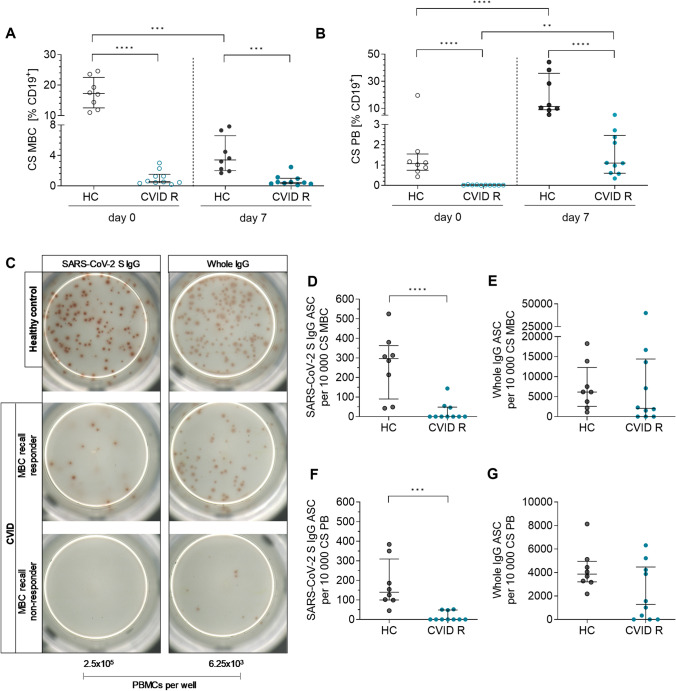
Impaired SARS-CoV-2 memory B cell recall response in CVID R patients after COVID-19 vaccination. Memory B cell (MBC) response was analyzed using a combination of flow cytometry to identify B cell subpopulations and ELISPOT assay for capacity of MBC to differentiate into antibody secreting cells (ASC). **A** Class-switched (CS) MBC and **B** CS PB frequencies assessed by flow cytometry ex vivo and after in vitro stimulation. **C** ELISPOT plate of SARS-CoV-2 MBC recall response and whole IgG positive control exemplarily shown for a HC and a CVID MBC recall responder and a CVID MC recall non-responder. SARS-CoV-2 S IgG ELISPOTS calculated per 10,000 CS MBC (**D**) and 10,000 CS PB (**F**). Whole IgG positive control for 10,000 CS MBC (**E**) and 10,000 CS PB (**G**). Unpaired comparisons across two groups were done by two-tailed non-parametric Mann–Whitney *U* test; paired comparisons within a group were done using the Wilcoxon matched pairs signed-rank test. *p* ≤ 0.05; **: *p* ≤ 0.01; ***: *p* ≤ 0.001; ****: *p* ≤ 0.0001

Based on flow cytometry staining at day 0 ex vivo and day 7 after in vitro stimulation using the SAC protocol, a decrease in both percentage and count of class-switched (CS) MBC was observed in HC (Fig. 2A). As expected, CVID R patients had initial significantly lower levels of CS MBC compared to HC (*p* < 0.0001). After expansion, CVID R patients elicited again significantly lower levels of CS MBC compared to HC (*p* = 0.003, Fig. 2A). CVID R patients also had significantly lower frequencies of CS PB ex vivo (*p* < 0.0001), and CS PB after in vitro stimulation (*p* < 0.0001, Fig. 2B). The decrease in frequency of MBC and increase in PB in HC indicates successful differentiation after in vitro stimulation. MBC of CVID R patients were also able to differentiate into PB following SAC stimulation, but to a much lower extent.

#### Deficient Memory B Cell Recall Response in Seropositive CVID Patients After COVID-19 Vaccination

Only 3/10 CVID R patients showed a minimal SARS-CoV-2 MBC recall response in the ELISPOT assay, whereas all HC showed a response (see Fig. 2C for exemplarily ELISPOT wells). CVID R patients had significantly lower SARS-CoV-2 ASCs per 10,000 CS MBC for S specific IgG (*p* = 0.0007) compared to HC (Fig. 2D). Regarding CS PB, ELISPOT elucidated a lower MBC recall response for S IgG (*p* = 0.001) in CVID R compared to HC as well (Fig. 2F). Whole IgG positive controls elicited high levels of ASCs in HC. Of note, whole IgG control responses were as well detectable in 7/10 CVID R patients. After normalizing spots per 10,000 CS PB and 10,000 CS MBC, counts did not differ significantly between the groups (*p* = 0.1 and *p* = 0.4 respectively, Fig. 2E and G).

### SARS-CoV-2 T Cellular Immune Response Is Robustly Induced in CVID Patients After COVID-19 Vaccination

In addition to humoral immune responses, the T cellular response was examined to evaluate if a SARS-CoV-2-specific T cell response is induced as a result of COVID-19 vaccination. Seroresponsive, non-seroresponsive CVID patients and HCs were comparatively analyzed.

#### Quantitative IFNγ Release by SARS-CoV-2-Specific T Cells

The IGRA enables the quantitative determination of IFNγ release by SARS-CoV-2 T cells after pathogen-specific stimulation. IGRA revealed a positive response in 6 CVID R and 3 CVID NR patients. One patient of each group was borderline positive. Negative results were obtained from 3 CVID R and 2 CVID NR patients. Stimulation with PMA served as positive control and showed positive results in all CVID patients despite one of the seroresponding individuals, which in contrast had a positive SARS-CoV-2-specific response (Table 4).

**Table 4:** Post COVID-19 vaccination IFNγ-release assay of SARS-CoV-2 peptide and PMA stimulated whole blood in CVID seroresponder and CVID non-seroresponder

ID	SARS-CoV-2 IFNγ [mIU/ml]	Mitogen control PMA IFNγ [mIU/ml]
CVID R 1	79.80	2403.20
CVID R 2	64.32	511.42
CVID R 3	147.15	2396.39
CVID R 4	677.59	2488.77
CVID R 5	227.29	2421.58
CVID R 6	618.80	44.74
CVID R 7	2483.27	2483.27
CVID R 8	202.30	2484.13
CVID R 9	2435.33	2435.33
CVID R 10	23.88	860.71
Median CVID R 1–10	214.78	2412.39
CVID NR 1	56.20	358.26
CVID NR 2	124.73	2306.53
CVID NR 3	466.27	2499.50
CVID NR 4	2499.50	2499.50
CVID NR 5	1128.41	938.55
CVID NR 6	34.58	2308.46
Median CVID NR 1–6	295.50	2307.50

*CVID*, common variable immunodeficiency disorder; *mIU/ml*, milli-international units per milliliter; *NR*, non-seroresponder; *R*, seroresponder; *rec.*, recurrent, *PMA*, Phorbol-12-myristat-13-acetat

#### SARS-CoV-2 Spike Reactive Polyfunctional T Cell Responses

SARS-CoV-2 S peptide–activated T cell subsets were assessed by flow cytometry. Antigen-specific CD4^+^ T cells were investigated using activation markers CD137 and CD154 along with expression of cytokines IFNγ, TNFα, and IL-2. An activated T cell response was defined as > 0.005% of total CD4^+^ T cells and 20% above the background signal. Polyfunctional cytokine subsets were obtained by Boolean combination gating. Moreover, formation of T_FH_ cells was investigated by staining of CXCR5 in activated CD4^+^ T cells (for gating, see Supplementary Fig. S2).

**Fig. 3:**
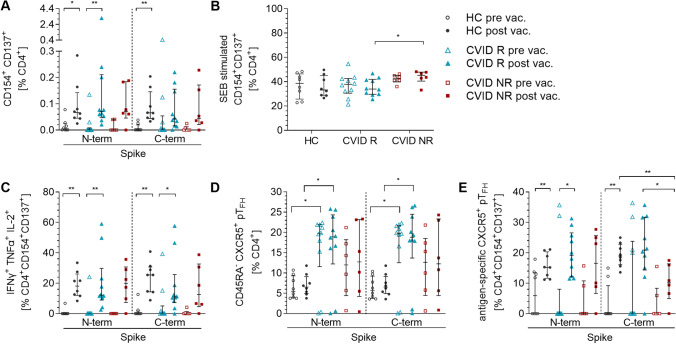
SARS-CoV-2 specific T cell responses in CVID patients and HC before and after COVID-19 vaccination. PBMCs were stimulated with 1 µg of SARS-CoV-2 S peptide pools or 3 µg SEB. Activated CD4^+^ T cell subsets were analyzed by multicolored flow cytometry. CD4^+^CD154^+^CD137^+^ T cells stimulated with SARS-CoV-2 S peptide pools (**A**) and SEB (**B**). Polyfunctional (IFNγ^+^TNFα^+^IL-2^+^) SARS-CoV-2 reactive CD4^+^CD154^+^CD137^+^ T cells (**C**). Peripheral TFH subset derived from CD4^+^CD45RA^−^CXCR5^+^ (**D**) and from SARS-CoV-2 reactive CD4^+^CD154^+^CD137^+^CXCR5.^+^ T cells (**E**). Unpaired comparisons across multiple groups were done by the Kruskal–Wallis test with Dunn’s post-test for multiple comparisons; unpaired comparisons across two groups were done by two-tailed non-parametric Mann–Whitney *U* test; paired comparisons within a group were done using the Wilcoxon matched pairs signed-rank test. *p* ≤ 0.05; **: *p* ≤ 0.01; ***: *p* ≤ 0.001; ****: *p* ≤ 0.0001

SARS-CoV-2 S reactive CD4^+^CD154^+^CD137^+^ T cells were induced in all HCs (N-term *p* = 0.016; C-term *p* = 0.008) and CVID R (N-term *p* = 0.004) after COVID-19 vaccination. In the group of CVID NR patients, 5 of 6 were able to generate a CD4^+^ T cell response to a similar extent than the two other groups (Fig. 3A). SEB positive control revealed comparable levels of CD4^+^CD154^+^CD137^+^ T cells before and after vaccination among all groups investigated indicating an intact T cell response. CVID NR patients showed slightly higher levels of activated CD4^+^ T cells post vaccination after SEB stimulation compared to CVID R patients (*p* = 0.02) (Fig. 3B).

Vaccination in our infection naïve study cohort induced comparable frequencies of polyfunctional activated CD4^+^CD154^+^CD137^+^ T cells in all groups. IFNγ^+^TNFα^+^IL-2^+^ triple-positive (tp) SARS-CoV-2 S N- and C-terminal reactive T cells significantly increased post vaccination in HC and CVID R (N-term: HC *p* = 0.008, CVID R *p* = 0.002; C-term: HC *p* = 0.008, CVID R *p* = 0.04). In the group of CVID NR, tp-activated CD4^+^ T cells were induced in 5/6 patients. Post vaccination tp cytokine responses did not differ between the three groups (Fig. 3C).

#### COVID-19 Vaccination Induces Spike-Specific Circulating TFH Cells

Higher frequencies of CD4^+^CD45RA^−^CXCR5^+^ peripheral T_FH_ cells were observed in CVID patients than in HC (Fig. 3D; for gating, see Supplementary Fig. S2). Induction of antigen reactive CD4^+^CD154^+^CD137^+^CXCR5^+^ peripheral T_FH_ cells by the COVID-19 vaccine was detected in all three groups (Fig. 3E). Frequencies of T_FH_ cells significantly increased in response to stimulation with SARS-CoV-2 S N-terminal peptide pool in HC (*p* = 0.008) and CVID R (*p* = 0.02). In CVID NR, 5/6 patients showed an increase but did not reach statistical significance. Regarding stimulation with the C-terminal peptide, pool frequencies were higher in HC after vaccination (*p* = 0.008). Moreover, SARS-CoV-2 S C-terminal reactive pT_FH_ were higher in HC compared to CVID NR (*p* = 0.008) as well as in CVID R compared to CVID NR (*p* = 0.02). Moreover, levels of SARS-CoV-2 spike–specific pT_FH_ cells did not correlate with antibody levels, antibody avidity or frequency of SARS-CoV-2 spike reactive polyfunctional CD4^+^ cells (see Supplementary Fig. S3).

## Discussion

Evaluation of humoral COVID-19 vaccine response revealed variable and in part surprisingly high rates of seroresponders among CVID patients. However, in a disease, which is defined by impaired antibody and B cell memory formation, quality and longevity of humoral immune response need to be considered.

In the present study, SARS-CoV-2 seroresponding and non-responding CVID patients as well as HC were comparatively analyzed for their antibody avidity and for the functional longevity of their humoral immune response by using a SARS-CoV-2 spike–specific MBC ELISPOT assay. In addition, T cellular immune response, including flow cytometric detection of SARS-CoV-2 reactive polyfunctional CD4^+^ T cells and T_FH_ cells, was assessed.

Despite detectable SARS-CoV-2 antibodies after two COVID-19 vaccinations, humoral immune response in CVID patients differed substantially from healthy individuals as SARS-CoV-2 spike antibodies in CVID patients showed a significantly reduced avidity in comparison to HC. Our study complements recently published data on avidity in CVID patients after COVID-19 vaccination [9, 16]. In contrast to our data, Sauerwein et al. observed similar levels of specific SARS-CoV-2 antibody levels and avidity in HC and CVID patients after two vaccinations, but reported lower avidity and antibodies after (3^rd^) booster vaccination [16]. Conflicting results may be due to different methodologies or might be related to the later time point of analysis and waning antibody levels. However, data from a kinetic study in CVID patients showed relatively stable anti-spike IgG antibody levels 4 weeks and 20 weeks after 2^nd^ COVID-19 vaccination and a positive trend for increased avidity after 3^rd^ vaccination [9]. Immunological and genetic heterogeneity within the group of COVID-19 seroresponding CVID patients are likely to contribute to the different observations.

SARS-CoV-2-specific IgG antibodies in peripheral blood are not informative about the source or MBC functionality and may arise from short- or long-lived plasma cells or from MBC after differentiation into ASC.

While conventional ELISPOT assay provides a qualitative and quantitative readout and can be designed to detect specific antibody responses [23, 24], the use of ELISPOT following in vitro stimulation and differentiation of MBC into ASC enables a functional analysis of specific B cell memory. Using an in-house SARS-CoV-2 spike–specific MBC ELISPOT assay, all HC showed a detectable response; however, only 3 (30%) seroresponding CVID patients had minimally detectable SARS-CoV-2 spike–specific IgG from ASCs after in vitro simulation and differentiation. Ratio of specific ASC per CS MBC as well as per CS PB was as significantly lower in CVID patients, indicating that the majority of seroresponding CVID patients failed to develop a robust humoral memory response.

Data on B cell memory in CVID patients after COVID-19 vaccination are very limited. Using flow cytometry, SARS-CoV-2–specific atypical MBC (defined as CD19^+^CD24^−^CD27^−^CD38^−^) with proposed low affinity were reported [17]. The present study provides additional functional data, showing an impaired specific recall memory response in seroresponding CVID patients. In the general population, SARS-CoV-2 mRNA-based vaccination induces both, a persistent germinal center (GC) B cell response and a robust but transient extra-follicular (EF) immune response resulting in antibodies of lower affinity from circulating PB [25, 26]. Lower avidity and impaired humoral memory formation argue for a predominantly EF and impaired GC response in our cohort of COVID-19-vaccinated CVID patients.

In addition to B cell differentiation and maturation, GC reaction involves multiple B cell extrinsic factors including specific (follicular) T cell interactions. Previous data suggested a correlation between reduced specific humoral immune response and impaired specific T cellular immunity in CVID patients [17, 27]. Our findings contrast this observation, with all CVID seroresponder and 5/6 non-seroresponder showing a robust polyfunctional CD4^+^ T cell immune response thus complementing previous reports of robust specific SARS-CoV-2 T cell responses in CVID patients with mild [28] and severe SARS-CoV-2 infections [29] as well as to COVID-19 vaccination [8–10] and other vaccines, such as influenza [30, 31]. A limitation of our study is the relatively long period of collecting samples in CVID patients and HC. While healthy control was analyzed earlier, all participating individuals were evaluated at least 4 weeks after 2^nd^ vaccination. Multiple studies show a stable specific SARS-CoV-2 CD4 + cellular immune response between 2 weeks and 6 months after second COVID-19 vaccination in healthy individuals [9, 32, 33] and for CVID patients [8].

The relatively broad range in specific T cell immunity in CVID patients may at least partly attributable to the applied methodology of analyzing SARS-CoV-2-specific T cell immunity. This is exemplified in our cohort by the variability of T cell responses ranging from 31 to 44% no or low responders when using commercially available IGRA assay and reaching 94% patients with polyfunctional triple-positive activated T cells by flow cytometry. In addition to methodological aspects, the general clinical and immunological heterogeneity of CVID patients may help to reconcile different observations. Higher frequencies of activated T cells in non-seroresponding CVID patients to SEB as positive control challenge the hypothesis of a generally impaired T cell immunity leading to a lower specific T cell response in our cohort of CVID patients [13].

T_FH_ cell and B cell interaction during GC reaction are a prerequisite for high-affinity antibody formation and levels of specific T_FH_ were reported to correlate positively with vaccine-induced antibodies against conjugated pneumococcal, hepatitis B, and influenza [34, 35]. In SARS-CoV-2, mRNA vaccination was shown to induce a robust specific T_FH_ response with stable persistence for at least 6 months after 2^nd^ vaccination [36].

The role of T_FH_ cells in CVID patients remains poorly understand. While a preserved T_FH_ response was observed upon influenza vaccination [31], lower levels of specific T_FH_ cells were reported in a cohort of COVID-19-vaccinated CVID patients [13]; however, analysis of T_FH_ cell response was not differentiated into seroresponding and seronegative patients in this study. In our cohort, we could successfully identify a T_FH_ response after stimulation with SARS-CoV-2 spike peptide pools with CVID seroresponder and HC expressing similar frequencies. However, in seronegative CVID patients, T_FH_ cells did not increase significantly after stimulation with N-terminal spike and also T_FH_ response to C-terminal SARS-CoV-2 spike peptide pool was significantly lower in seronegative than in seroresponding CVID patients. This observation suggests an important role of T_FH_ cells during COVID-19 vaccine response. However, we did not observe a correlation between levels of specific T_FH_ cells with antibody levels or avidity.

Higher levels of activated CXCR5^+^ peripheral T_FH_ cells were reported previously for CVID patients [37] in particular in patients with non-infectious manifestations (autoimmunity and granulomatous disease), suggesting a functional significance of this association. However, T_FH_ cells form a functionally and phenotypically heterogeneous group. Although high expression of CXCR5 is one of the defining hallmarks of T_FH_, CXCR5 is also expressed on 20–25% of peripheral blood human central memory CD4^+^ T cells [38]. Detection of this subgroup prior to SARS-CoV-2 spike stimulation may therefore represent an unspecific state of activation. Of note, in CVID patients, peripheral T_FH_ cells with phenotypical markers of activation were recently shown to express an mRNA signature of exhaustion, apoptosis, and senescence [39]. These observations should prompt caution when interpreting phenotypical findings in the context of T_FH_ functionality. To further understand the role of T_FH_ cells would require a detailed analysis, including functional assays and an expanded marker profile, in larger cohorts. Given the heterogeneity of changes in GC reactions in CVID patients, a uniform pathomechanism of impaired COVID-19 vaccine response is unlikely [40–44].

Limitations of our study include the slight heterogeneity and timing of administered COVID-19 vaccines and that interpretation is restricted to patients after receiving two COVID-19 vaccinations. While more than two vaccinations were shown to increase humoral immune response in CVID patients [18], it remains uncertain whether repeated vaccinations could also translate into B cell memory formation. Due to increased levels of SARS-CoV-2-IgG antibodies in commercially available immunoglobulins [45], a mere serological evaluation does no longer allow to distinguish between passive immunization and active antibody generation. Using SARS-CoV-2-specific MBC ELISPOT assays may enable researchers to circumvent this uncertainty. HC were significantly younger; however by providing data before and after vaccination, we were able to analyze comparatively intra-individual responses within each group.

The identification of possible predictive factors of a strong or impaired immune response to COVID-19 vaccination would improve risk stratification and support an individual prophylactic management for CVID patients. Previous vaccination studies in CVID patients described the potential impact of the type of vaccine and distribution of B cell subsets affecting vaccination responses [46]. Regarding COVID-19 vaccine response in CVID, a range of immunological and clinical factors have been described, including non-infectious complications and ongoing immunosuppressive therapy as well as elevated CD21^low^ B cells, low B cells, low naïve T cells, and reduced IgA and IgM levels [7, 11, 47]. In our cohort, CVID seroresponder and non-seroresponder did not differ in any key immunological parameter; however the small number of patients limits the interpretation. Of note, and in line with recent observations [9], previous failure to mount a specific antibody response to pneumococcal conjugate vaccine could not predict COVID-19 vaccine response in our cohort of CVID patients. This discrepancy might be related to the antigen structure, but immunogenicity might also depend on the mode of immunization (mRNA vs conjugated vaccine) [48]. While humoral immune response to pneumococcal conjugated vaccine requires GC reaction, response to COVID-19 involves both GC and EF structures [49]. Immunological and genetic aspects that shape the humoral immune response to SARS-CoV-2 vaccination in CVID patients remain incompletely understood. However, the observation of seroresponding and non-seroresponding CVID patients may help to shed light onto the diverse pathomechanisms of CVID. Further studies in larger cohorts are required to evaluate possible underlying B cell differentiation defects in CVID patients.

## Conclusions

Reduced avidity of SARS-CoV-2 IgG and significantly impaired recall memory formation after COVID-19 vaccination in seroresponding CVID patients stress the importance of a more differentiated analysis of humoral immune response in CVID patients. Our observations challenge the binary categorization into seroresponder and non-seroresponder and potentially impact on clinical decisions for the prophylactic management of COVID-19.

## Supplementary Information

Below is the link to the electronic supplementary material.

Supplementary file1 (DOCX 510 KB)

## Data Availability

The datasets generated during and/or analyzed during the current study are available from the corresponding author on reasonable request.
