# Supplementary material for: Impaired B Cell Recall Memory and Reduced Antibody Avidity but Robust T Cell Response in CVID Patients After COVID-19 Vaccination
Source: J Clin Immunol. 2023 Mar 17;43(5):869–81. doi: 10.1007/s10875-023-01468-w (PMC10023009; doi:10.1007/s10875-023-01468-w)
Supplement: Supplementary file 1 — Supplementary file1 (DOCX 510 KB) [file 10875_2023_1468_MOESM1_ESM.docx]

**Supplementary Tables**

**Table S1:** Cohort Characteristics for healthy controls and CVID patients

| **ID** | **Age** | **Gender** | **Ethnicity** | **Administered vaccine** |
| --- | --- | --- | --- | --- |
| HC-1 | 34 | f | caucasian | ChAdOx1-S / BNT162b2 |
| HC-2 | 39 | f | caucasian | ChAdOx1-S / BNT162b2 |
| HC-3 | 44 | m | caucasian | ChAdOx1-S / BNT162b2 |
| HC-4 | 35 | f | caucasian | ChAdOx1-S / BNT162b2 |
| HC-5 | 39 | f | caucasian | ChAdOx1-S / BNT162b2 |
| HC-6 | 30 | f | caucasian | 2 x BNT162b2 |
| HC-7 | 34 | m | caucasian | ChAdOx1-S / BNT162b2 |
| HC-8 | 28 | m | caucasian | 2 x mRNA-1273 |
| Median | 35 |  |  |  |
|  |  |  |  |  |
| **ID** | **Age** | **Gender** | **Ethnicity** | **Administered vaccine** |
| CVID R 1 | 41 | f | caucasian | 2 x BNT162b2 |
| CVID R 2 | 42 | f | caucasian | 2 x BNT162b2 |
| CVID R 3 | 62 | f | caucasian | 2 x BNT162b2 |
| CVID R 4 | 56 | m | caucasian | 2x ChAdOx1-S |
| CVID R 5 | 32 | f | caucasian | ChAdOx1-S / mRNA-1273 |
| CVID R 6 | 62 | f | caucasian | 2 x BNT162b2 |
| CVID R 7 | 49 | f | caucasian | 2 x BNT162b2 |
| CVID R 8 | 62 | f | caucasian | 2 x ChAdOx1-S |
| CVID R 9 | 59 | m | caucasian | 2 x BNT162b2 |
| CVID R 10 | 62 | m | caucasian | 2 x BNT162b2 |
| Median | 57 |  |  |  |
| CVID NR 1 | 63 | m | caucasian | 2 x BNT162b2 |
| CVID NR 2 | 54 | f | caucasian | ChAdOx1-S / BNT162b2 |
| CVID NR 3 | 48 | m | caucasian | 2 x BNT162b2 |
| CVID NR 4 | 54 | m | caucasian | ChAdOx1-S / BNT162b2 |
| CVID NR 5 | 31 | f | caucasian | 2 x BNT162b2 |
| CVID NR 6 | 69 | f | caucasian | 2 x BNT162b2 |
| Median | 57 |  |  |  |

CVID = common variable immunodeficiency disorder; f = female; m = male; NR = non-seroresponder; R = seroresponder

**Table S2:** Serological data of healthy controls and CVID patients after COVID-19 vaccination (SeraSpot©)

| **after COVID-19 vaccination** | | | | | | |
| --- | --- | --- | --- | --- | --- | --- |
|  | **Ratio NP IgG** | **Ratio RBD IgG** | **Ratio S1 IgG** | **Ratio S full IgG** | **Anti-SARS-CoV-2 NP IgG** | **Anti-SARS-CoV-2 Spike IgG** |
| **CVID R 1** | 0.05 | 7.77 | 5.59 | 6.64 | negative | reactive |
| **CVID R 2** | 0.11 | 4.7 | 3.22 | 3.97 | negative | reactive |
| **CVID R 3** | 0 | 4.56 | 3.32 | 3.32 | negative | reactive |
| **CVID R 4** | 0 | 2.5 | 1.57 | 2.0 | negative | reactive |
| **CVID R 5** | 0.05 | 1.43 | 1.1 | 1.37 | negative | reactive |
| **CVID R 6** | 0.06 | 4.56 | 2.66 | 3.59 | negative | reactive |
| **CVID R 7** | 0.07 | 7.07 | 5.87 | 6.47 | negative | reactive |
| **CVID R 8** | 0.08 | 3.64 | 3.08 | 3.11 | negative | reactive |
| **CVID R 9** | 0.06 | 5.88 | 4.59 | 5.32 | negative | reactive |
| **CVID R 10** | 0 | 3.86 | 2.36 | 2.64 | negative | reactive |
|  | | | | | | |
| **CVID NR 1** | 0.11 | 0.97 | 0.47 | 0.67 | negative | negative |
| **CVID NR 2** | 0 | 0.31 | 0.08 | 0.19 | negative | negative |
| **CVID NR 3** | 0.08 | 0.03 | 0 | 0.03 | negative | negative |
| **CVID NR 4** | 0.2 | 0.1 | 0 | 0.17 | negative | negative |
| **CVID NR 5** | 0.39 | 0.19 | 0.03 | 0.23 | negative | negative |
| **CVID NR 6** | 0.08 | 0 | 0 | 0 | negative | negative |
|  | | | | | | |
| **HC-1** | 0.03 | 6.29 | 5.86 | 5.89 | negative | reactive |
| **HC-2** | 0.05 | 5.95 | 5.57 | 5.73 | negative | reactive |
| **HC-3** | 0 | 6.09 | 5.29 | 5.53 | negative | reactive |
| **HC-4** | 0.06 | 6.14 | 5.29 | 5.6 | negative | reactive |
| **HC-5** | 0.14 | 5.19 | 4.69 | 4.98 | negative | reactive |
| **HC-6** | 0.17 | 7.13 | 6.43 | 6.47 | negative | reactive |
| **HC-7** | 0.1 | 5.39 | 5.2 | 5.12 | negative | reactive |
| **HC-8** | 0 | 5.94 | 4.83 | 5.17 | negative | reactive |

COVID-19 = coronavirus disease 2019; CVID = common variable immunodeficiency disorder; HC = healthy control; NP = nucleocapsid protein; NR = non-seroresponder OD = optical densitiy; R = seroresponder; RBD = receptor binding domain; S = Spike; S1 = S1 subunit of spike protein

**Table S3:** Marker for T cell phenotyping

| Target | Conjugate | Clone | Dilution | Company |
| --- | --- | --- | --- | --- |
| Anti-human CD3 | BV650 | OKT3 | 1:100 | Biolegend |
| Anti-human CD4 | PerCp-Cy5.5 | SK3 | 1:100 | Biolegend |
| Anti-human CD8 | BV510 | RPA-T8 | 1:100 | Biolegend |
| Anti-human CD137 | PE | 4B4-1 | 1:100 | Biolegend |
| Anti-human CD154 | BV421 | 24-31 | 1:200 | Biolegend |
| Anti-human IL-2 | APC | MQ1-17H12 (RUO) | 1:200 | BD |
| Anti-human IFNγ | BV605 | 4S.B3 | 1:20 | Biolegend |
| Anti-human TNFα | AF700 | MAb11 | 1:20 | Biolegend |
| Anti-human CCR7 | AF488 | G043H7 | 1:100 | Biolegend |
| Anti-human CD45-RA | PE-Cy7 | HI100 | 1:100 | Biolegend |
| Anti-human CXCR5 | PE-Dazzle | J252D4 | 1:100 | Biolegend |
| Dead Cell Staining | Fixable Blue |  | 1:100 | Thermo Fisher |

**Table S4:** Marker for B cell and plasmablast phenotyping

| Target | Conjugate | Clone | Dilution | Company |
| --- | --- | --- | --- | --- |
| Anti-human CD3 | Pacific Blue | UCHT1 | 1:50 | Biolegend |
| Anti-human CD19 | PE-Cy7 | HIB19 | 1:66 | Biolegend |
| Anti-human CD21 | PE | Bu32 | 1:50 | Biolegend |
| Anti-human CD24 | PerCp-Cy5.5 | ML5 | 1:66 | Biolegend |
| Anti-human CD27 | FITC | M-T271 | 1:40 | Biolegend |
| Anti-human CD38 | Alexa Fluor 700 | HIT2 | 1:50 | Biolegend |
| Anti-human IgM | APC | MHM-88 | 1:40 | Biolegend |
| Anti-human IgD | APC-Cy7 | IA6-2 | 1:50 | Biolegend |
| Dead Cell Staining | Fixable Aqua |  | 1:50 | Thermo Fisher |

**Table S5:** Subsets of B cells and plasmablasts within single, living CD3^-^CD19^+^ lymphocytes

| B cell subset | Marker |
| --- | --- |
| Naïve B cells | IgD^+^CD27^-^ |
| Marginal zone like B cells (MZ-like) | IgD^+^CD27^+^ |
| Memory B cells | IgD^-^CD27^+^ |
| IgM only memory B cells | IgD^-^CD27^+^IgM^+^ |
| Class-switched memory B cells | IgD^-^CD27^+^IgM^-^ |
| Plasmablasts | CD38^++^CD27^++^IgD^-^ |
| IgM only Plasmablasts | CD38^++^CD27^++^IgD^-^IgM^+^ |
| Class-switched Plasmablasts | CD38^++^CD27^++^IgD^-^IgM^-^ |

**Supplementary Figure S1:** Gating strategy for B cell phenotyping.


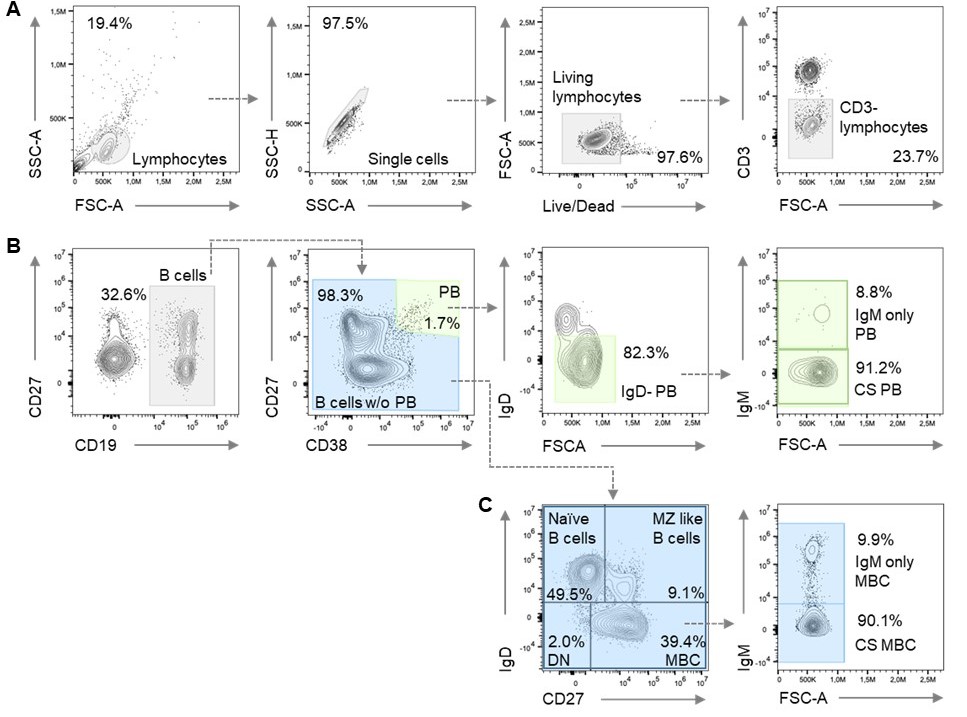


**Supplementary Figure S2:** Gating strategy for SARS-CoV-2 spike-specific T cell subsets and their cytokine expression.


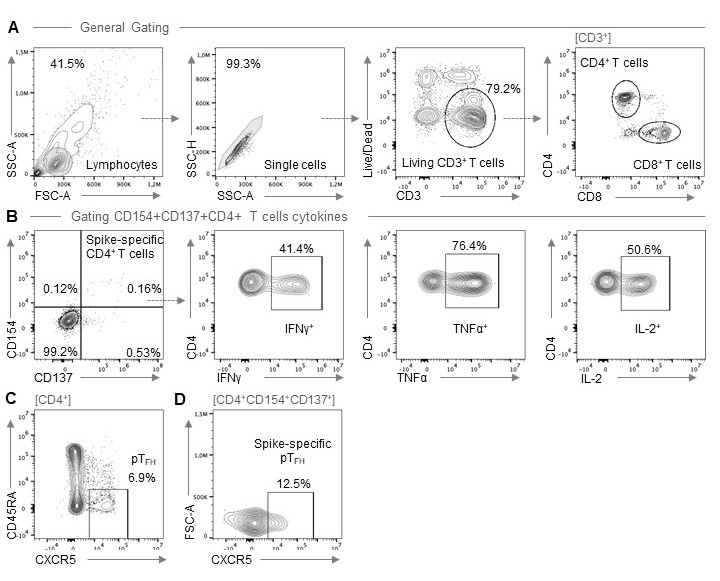


**Supplementary Figure S3:** Correlation of SARS-CoV-2 antibody avidity and SARS-CoV-2 spike reactive CD4^+^CD154^+^CD137^+^CXCR5^+^ peripheral T_FH_ cells in COVID-19 vaccinated CVID patients and healthy controls.
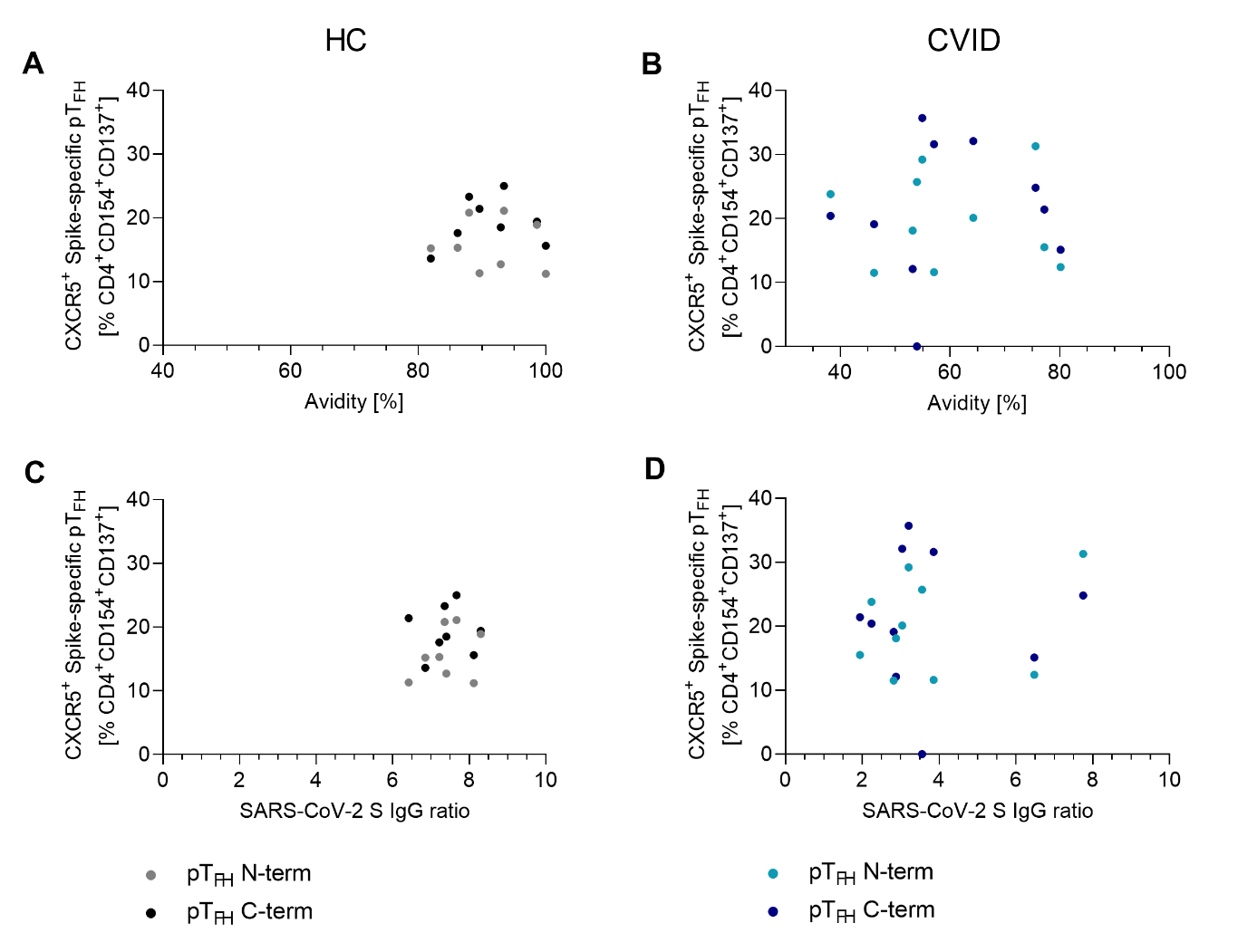


**Online Resource Legends**

**Supplementary Figure S1: Gating strategy for B cell phenotyping.** Gating is exemplary shown in thawed PBMCs of a healthy individual in *ex vivo* condition. **(A)** Strategy for analyzing B cell subsets in PBMCs starting from lymphocytes followed by exclusion of duplets together with dead cells and selection of CD3^-^ lymphocytes. **(B)** Strategy for assessment of plasmablasts (PB) derived from CD19^+^ B lymphocytes. PBs are defined as CD38^++^CD27^++^. Following identification of PB. cells were gated on IgD^-^ cells and their expression of IgM to identify IgM only and class-switched (CS) PB. **(C)** Gating strategy for B cells without PB to analyze and IgD^-^CD27^+^ memory B cells (MBC) and their expression of IgM for IgM only and CS MBC.

**Supplementary Figure S2: Gating strategy for SARS-CoV-2 Spike-specific T cell subsets and their cytokine expression.** Gating is exemplary shown for a healthy individual after peptide stimulation. **(A)** Strategy for analyzing T cell subsets in PBMCs starting from lymphocytes followed by exclusion of duplets together with dead cells and selection of CD3^+^ T cells. which were further subdivided into CD4^+^ and CD8^+^ T cells. **(B)** CD4^+^ T cells were further gated on CD154^+^CD137^+^ activated T cells and their expression of IFNγ. TNFα and IL-2. **(C)** Gating of CD45RA^-^CXCR5^+^ pT_FH_ cells derived from CD4^+^ T cells. **(D)** Gating of SARS-CoV-2 Spike specific CXCR5^+^ pT_FH_ cells derived from CD4^+^CD154^+^CD137^+^ T cells.

**Supplementary Figure S3: Correlation of SARS-CoV-2 antibody avidity and SARS-CoV-2 spike reactive CD4^+^CD154^+^CD137^+^CXCR5^+^ peripheral T_FH_ cells in COVID-19 vaccinated CVID R patients and healthy controls. (A-B)** Correlation analysis of SARS-CoV-2 spike-specific pT_FH_ with SARS-CoV-2 S IgG antibody ratio in HC **(A)** and CVID R patients **(B)** and of SARS-CoV-2 Spike-specific pT_FH_ with antibody avidity in HC **(C)** and CVID R patients **(D)**. Correlation analysis was performed using Spearman’s rank correlation coefficient.

**Table S1:** Cohort characteristics for healthy controls and CVID patients

**Table S2:** Serological data of CVID patients after COVID-19 vaccination (SeraSpot©)

**Table S3:** Marker for T cell phenotyping

**Table S4:** Marker for B cell and plasmablast phenotyping

**Table S5:** Subsets of B cells and plasmablasts within single, living CD3^-^CD19^+^ lymphocytes
